# Dysregulated long intergenic non-coding RNA modules contribute to heart failure

**DOI:** 10.18632/oncotarget.10834

**Published:** 2016-07-25

**Authors:** Lin Pang, Jing Hu, Guanxiong Zhang, Xiang Li, Xinxin Zhang, Fulong Yu, Yujia Lan, Jinyuan Xu, Bo Pang, Dong Han, Yun Xiao, Xia Li

**Affiliations:** ^1^ College of Bioinformatics Science and Technology, Harbin Medical University, Harbin, Heilongjiang, China; ^2^ Department of Genetics, Harbin Medical University, Harbin, Heilongjiang, China; ^3^ National Center for Nanoscience and Technology, Haidian, Beijing, China; ^4^ Key Laboratory of Cardiovascular Medicine Research, Harbin Medical University, Ministry of Education, Harbin, Heilongjiang, China

**Keywords:** lincRNAmodule, heart failure, ceRNA, contraction, heart specificity

## Abstract

Long intergenic non-coding RNAs (lincRNAs) are emerging as important regulatory molecules involved in diseases including heart failure. However, little is known about how the lincRNAs work together with protein-coding genes (PCGs) contributing to the pathogenesis of heart failure. In this study, we constructed a comprehensive transcriptome profile of lincRNAs, PCGs and miRNAs using RNA-seq and miRNA-seq data of 16 heart failure patients (HFs) and 8 non-failing individuals (NFs). Through integrating lincRNA and PCG expression profiles, we identified HF-associated lincRNA modules. We identified a heart-specific lincRNA module which was significantly enriched for differentially expressed lincRNAs and PCGs. This module was associated with heart failure rather than with other clinical traits such as sex, age, smoking and diabetes mellitus. Moreover, the module was significantly correlated with certain indicators of left ventricular function like ejection fraction and left ventricular end-diastolic diameter, implying the potential of its components as crucial biomarkers. Apart from enhancer-like function, lincRNAs in this module could act as competing endogenous RNAs (ceRNAs) to regulate genes which were associated with left-ventricular systolic function. Our work provided deep insights into the critical roles of lincRNAs in the pathology of heart failure and suggested that they could be valuable biomarkers and therapeutic targets.

## INTRODUCTION

Heart failure (HF) is the most devastating cardiovascular disease with high morbidity and mortality affecting approximately 38 million patients worldwide, and the number increases substantially with the ageing of the population [1]. A global transcriptional reprogramming is thought to be the base of pathological HF, rendering the reactivation of developmental cardiac gene program [2]. Over the past several decades, great efforts have been made to characterize the transcriptomes of HF extensively. As a consequence, a number of potential biomarkers and therapeutic targets have been identified [3, 4]. However, although these protein-centric biomarkers and therapeutic targets have shown, to some extent, to be of benefit for the diagnosis and treatment of HF, the prognosis of HF is still poorer than that of most cancers [1].

Most of the human genome can be transcribed while only 2% code for proteins, thus producing large numbers of non-coding RNAs (ncRNAs) [5]. Currently, the best-characterized ncRNAs in the heart are the microRNAs (miRNAs), which are involved in the pathophysiologic aspects of HF, such as miR-1, miR-133 and miR-132 [6]. In addition to miRNAs, long intergenic non-coding RNAs (lincRNAs) have also attracted many researchers due to their implications in biological development and disease progression [6], where lincRNAs could function as molecular signals, decoys, guides, scaffolds and competing endogenous RNAs (ceRNAs) [7, 8]. The ceRNAs are pairs of genes that can regulate each other's expression through competing for common endogenous miRNAs, representing a miRNA-mediated post-transcriptional gene regulation. There have been several studies demonstrating their involvement in cardiac pathophysiology. For example, lincRNA *LIPCAR* was shown to be a novel biomarker of cardiac remodeling and could predict the survival of patients with HF [9]. Six single-nucleotide polymorphisms (SNPs) in the lincRNA *MIAT* were found to be associated with myocardial infarction by a genome-wide association study [10]. However, these studies were mostly focused on single lincRNAs while systematical characterization of lincRNAs in HF remain scarce.

In this study, we characterized transcriptome profiles of lincRNAs, mRNAs and miRNAs in heart failure using RNA-seq and miRNA-seq data. We demonstrated that lincRNAs and PCGs formed multiple modules to exert diverse functions in heart failure. Specifically, one module, which was most significantly enriched in differentially expressed lincRNAs, was highly heart-specific and showed tight correlation with ejection fraction and left ventricular end-diastolic diameter, suggesting potential biomarkers within it. Moreover, we demonstrated that lincRNAs and PCGs in this module could form ceRNA pairs to perform essential roles in heart failure, such as regulation of cardiac muscle contraction and regulation of actin filament–based movement.

## RESULTS

### Constructing transcriptome profiles of lincRNA, mRNA and miRNA in heart failure

**Figure 1:**
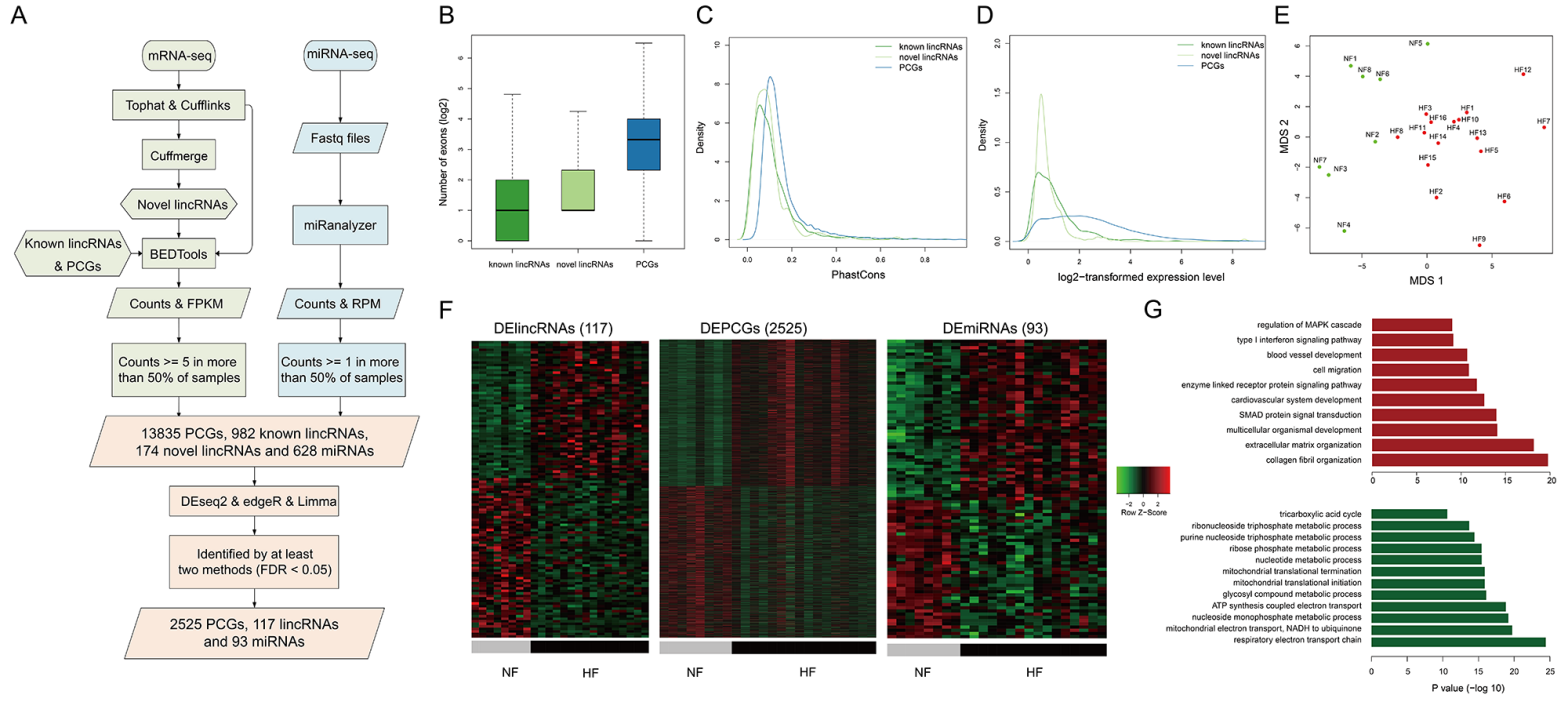
Transcriptome profiles of lincRNAs, mRNAs and miRNAs **A.** The workflow of the construction of transcriptome. The basic features of transcriptome about **B.** the numbers of exons, **C.** conservation, **D.** expression levels of known lincRNAs, novel lincRNAs and PCGs. **E.** MDS plot using the lincRNA expression levels. **F.** Heatmaps showing hierarchical clustering of differentially expressed genes and miRNAs. **G.** Functional enrichment results for up-regulated (red bars) and down-regulated PCGs (green bars).

We obtained RNA-seq and miRNA-seq data of 16 patients with heart failure (HFs) and 8 non-failing individuals (NFs) to construct transcriptome profiles of lincRNAs, mRNAs and miRNAs (Figure 1A, see Methods). The demographic details of the samples were summarized in Supplementary Table S1. For RNA-seq data, reads were mapped against the human genome using Tophat. Cufflinks and Cuffmerge were used to assemble and merge transcripts. For miRNA-seq data, miRanalyzer was used to detect annotated miRNAs based on miRBase v19. After filtering lowly-expressed genes and miRNAs, we identified 982 known lincRNAs, 174 novel lincRNAs, 13835 protein-coding genes (PCGs) and 628 miRNAs.

In order to validate the data used in our study, we collected another set of RNA-seq data from an independent cohort composed of 3 non-failing samples and 3 failing samples (GSE57344). As a result, we observed strong correlation of expression profiles between the data in our study and GSE57344 (Supplementary Figure S1A-S1C). Additionally, we examined the expression levels of three known heart failure-related lncRNAs, LIPCAR [9], MIAT [10] and ANRIL [11]. We observed significant expression changes of all these three lncRNAs in heart failure patients (Supplementary Figure S1D), further supporting the validity of the data.

We found that the exon numbers of lincRNAs were significantly smaller than those of PCGs (*P* value < 2.2e-16, Wilcoxon rank-sum test, Figure 1B). We calculated the exon conservation by phastCons algorithm [12] and found that both known and novel lincRNA exons were less conserved than coding exons (Figure 1C). Moreover, linRNAs showed significantly lower expression levels than PCGs (*P* value <2.2e-16, Wilcoxon rank-sum test, Figure 1D). All of lincRNAs, PCGs and miRNAs could distinguish HFs from NFs (Figures 1E and S2), suggesting distinct transcriptomic expression patterns between HFs and NFs.

To further characterize the transcriptional alterations in HF, differential expression analysis was performed through integrating DESeq2, edgeR and voom-limma (see Methods). Finally, we identified 117 differentially expressed lincRNAs (DELincRNAs, 54 up-regulated and 63 down-regulated), 2525 DEPCGs (1241 up-regulated and 1284 down-regulated) and 93 DEmiRNAs (50 up-regulated and 43 down-regulated) (Figure 1F). Functional enrichment analysis showed that up-regulated PCGs were involved in cardiovascular system development, blood vessel development and signaling pathways, while down-regulated PCGs were involved in energy and metabolic processes, such as tricarboxylic acid cycle and ATP synthesis (Figure 1G).

### Identifying lincRNA-PCG modules in heart failure

**Figure 2:**
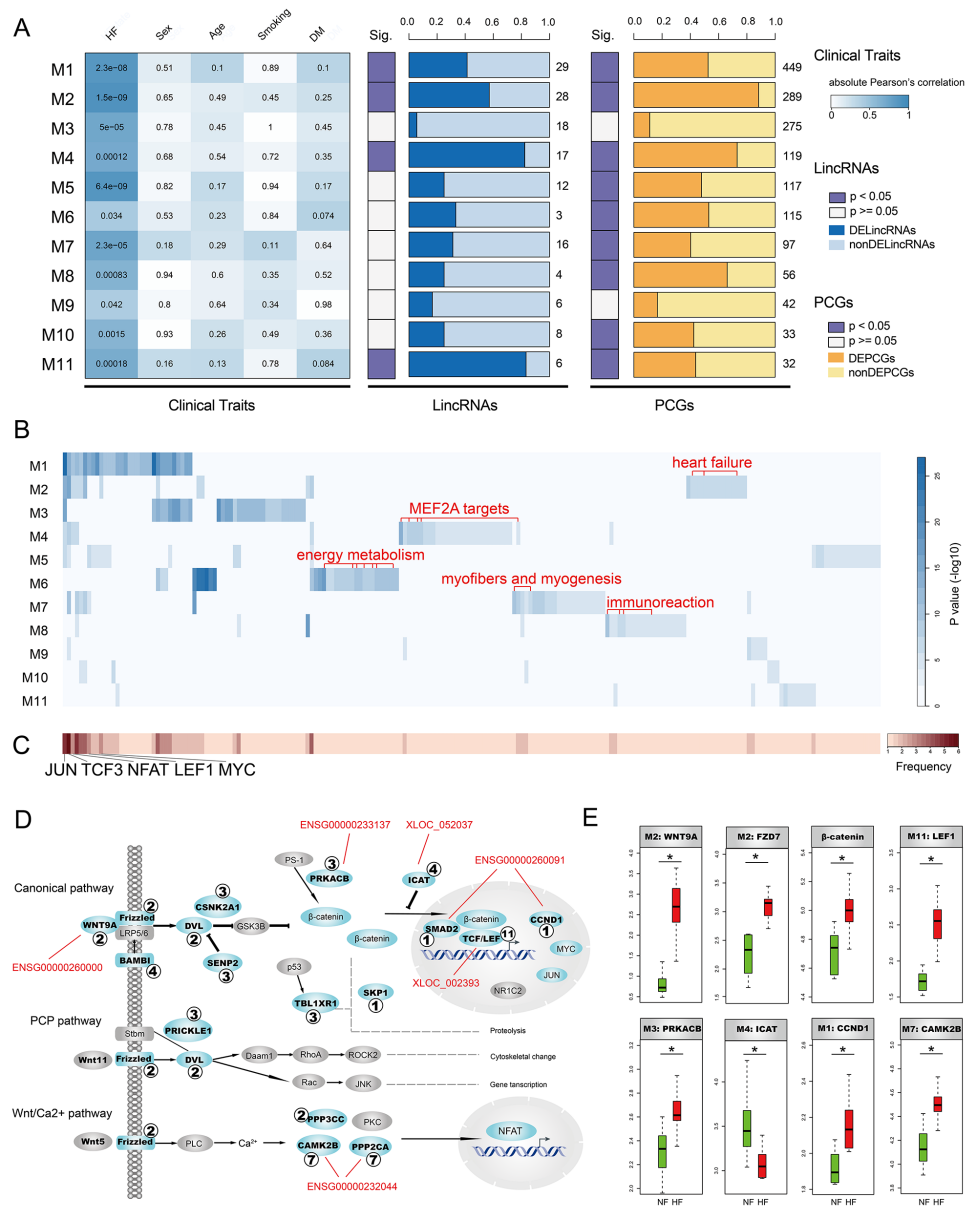
Identification and characterization of HF-associated lincRNA modules **A.** The heatmap on the left panel represents absolute Pearson's correlation between 11 modules and clinical traits including heart failure (HF), sex, age, smoking and diabetes mellitus (DM). P-values of correlation test were filled in the cells. The two panels on the right side denote proportions of differentially expressed lincRNAs and PCGs. The numbers denote the amount of lincRNAs or PCGs in the corresponding modules. **B.** Enrichment results of gene sets obtained from GSEA Molecular Signatures Database (MSigDB) for heart-associated lincRNA modules. Several module-specific functional gene sets are highlighted in red. **C.** Heatmap representing frequencies of gene sets enriched by heart-associated lincRNA modules. **D.** heart-associated lincRNA modules together participate in Wnt signaling pathway. Genes in modules are colored cyan with corresponding module IDs attached. Several lincRNAs highly co-expressed with important components of the Wnt signaling pathway are highlighted in red. **E.** Several important components in the Wnt signaling pathway show significant expression alterations in heart failure.

LincRNAs could form co-expression modules with PCGs to exert important functions [13, 14]. To explore contributions of lincRNAs to heart failure, we leveraged an unsupervised and unbiased approach WGCNA [15, 16] (see Methods) and identified 45 modules, of which 23 were associated with heart failure rather than with other clinical traits such as sex, age, smoking and diabetes mellitus (DM). Then we retained 11 HF-associated modules containing DELincRNAs with less than 500 genes. These modules were defined as HF-associated lincRNA modules, corresponding to 111 known lincRNAs, 36 novel lincRNAs and 1624 PCGs (Figure 2A). Among these modules, 4 were significantly enriched with both DELincRNAs and DEPCGs (hypergeometric test, *P* value < 0.05). Modules M4 and M11 contained the highest proportions of DELincRNAs, and M2 and M4 were the modules with the highest proportions of DEPCGs. We also revealed two modules (M12 and M13) with greater than 500 genes (see Supplementary Figure S3 for details about the two modules).

To investigate the biological functions of these HF-associated modules, we assessed the enrichment of 10348 functional gene set collections from the GSEA Molecular Signatures Database using hypergeometric test (FDR < 0.05, Figure 2B and Supplementary Figure S3B). We observed that several modules were enriched in some common gene sets which were potential targets of transcription factors JUN, TCF3, NFAT, LEF1 and MYC (Figure 2C). Of note, these transcription factors were important components of Wnt signaling pathway. We thus explored implications of these modules in the Wnt signaling pathway and found that six modules, including M1, M2, M3, M4, M7 and M11, participated in different parts of the Wnt signaling pathway (Figure 2D). It was suggested that the Wnt signaling pathway was triggered by binding of Wnt proteins to receptors of the Frizzled family [17]. We found that*WNT9A* and *FZD7* were up-regulated in HF (Figure 2E), indicating activation of the Wnt signaling pathway. Moreover, we also observed up-regulation of *PRKACB*, *β-catenin*, *LEF1* and *CCND1* and down-regulation of *ICAT*, further supporting this notion (Figure 2E). Consistently, there were several studies suggesting activation of Wnt signaling pathway in myocardial hypertrophy and remodeling [18–20]. Additionally, we found that several lincRNAs highly co-expressed (absolute Pearson's correlation coefficient >0.75) with critical components of the Wnt signaling pathway (Figure 2D), including *ENSG00000260000*, *ENSG00000233137*, *XLOC_052037*, *ENSG00000260091*, *XLOC_002393* and *ENSG00000232044*, all of which were up-regulated in HF except *ENSG00000232044*. These results suggested that lincRNAs and PCGs could form co-expression modules and these modules could together be implicated in activation of the Wnt signaling pathway with different modules playing roles in different parts.

Interestingly, we observed that different modules tended to be enriched in distinct functions (Figure 2B). For instance, several gene sets down-regulated in heart failure were only enriched by M2. Consistently, these genes were all significantly down-regulated in our HF cases compared to NF controls (Supplementary Figure S4), supporting the accuracy and reliability of our results. M4 was specifically enriched in gene sets harboring MEF2A motifs. Of note, MEF2A was one of the key cardiac transcription factors which played pivotal roles in the differentiation, maturation and homeostasis of cardiomyocytes [21]. Several energy metabolism-related gene sets, such as mitochondria genes and genes involved in the citric acid (TCA) cycle and respiratory electron transport, were only enriched by M6. It's known that energy metabolism is broadly changed to affect both cardiac and skeletal muscles in heart failure [22]. Similarly, myofibers and myogenesis-related gene sets were only enriched by M7 and immunoreaction-related gene sets were mainly enriched by M8 (Figure 2B). Taken together, HF-associated lincRNA modules were involved in important functions related to heart failure and different modules performed different functions.

### A heart-specific module correlates with indicators of left ventricular function

**Figure 3:**
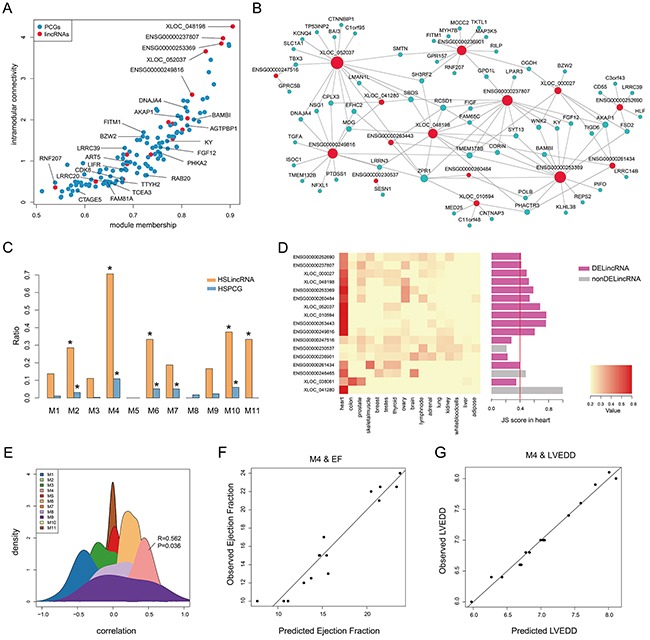
Heart specificity of M4 **A.** Connectivity between genes (lincRNAs and PCGs) in M4 is plotted against the module's first principal component. Red and blue dots represent lincRNAs and PCGs, respectively. **B.** Co-expression network of M4 in which a lincRNA is linked to a PCG if their expression correlation is greater than 0.75. **C.** Barplot of the ratio of TSLincRNAs and TSPCGs in each heart-associated lincRNA module. **D.** Heatmap showing hierarchical clustering of JS scores of lincRNAs in M4 and barplot of JS score of each lincRNA in M4. **E.** The density plot of Spearman correlation between lincRNAs and EF in each heart-associated lincRNA modules. **F.** Multiple correlation scatter plot of predicted EF and observed EF. **G.** Multiple correlation scatter plot of predicted LVEDD and observed LVEDD.

Since M4 was most significantly enriched for both DELincRNAs and DEPCGs, we further dissected M4 to assess its contribution to heart failure. M4 contained 136 genes including 17 lincRNAs and 119 PCGs, among which 14 lincRNAs and 87 PCGs were differentially expressed. To identify relatively more important genes in M4, we estimated the intramodular connectivity and correlation with the first principal component of the module for each gene and found *ENSG00000237807*, *ENSG00000249816*, *XLOC_052037* and *XLOC_048198* were among the highest-ranking genes (Figure 3A). Network analysis based on co-expression consistently revealed the important regulatory roles of lincRNAs in M4 (Figure 3B).

Given that M4 was enriched in potential targets of MEF2A (Figure 2B and 3A), a myocyte-specific transcription factor, we speculated M4 to be a heart-specific module. To validate this, we first downloaded RNA-seq data of 16 tissues from Human Body Map 2.0 and calculated the JS scores for all expressed genes to evaluate tissue specificity (see Methods). Hierarchical clustering based on JS scores of lincRNAs, rather than that of PCGs, could separate heart from other tissues, revealing a higher heart specificity for lincRNAs (Supplementary Figure S5). Using a cut-off of 0.4, we identified 132 heart-specific lincRNAs and 108 heart-specific PCGs in total. Furthermore, we calculated the ratios of heart-specific lincRNAs and PCGs in each HF-associated lincRNA module and observed that M4 showed the highest heart specificity with significant enrichment of heart-specific lincRNAs and PCGs (*P* value < 0.05, hypergeometric test, Figure 3C and Supplementary Figure S3C). Particularly, 12 (71%) lincRNAs in M4 were heart-specific, of which 10 were differentially expressed (Figure 3D). These findings implied a potentially strong association with cardiac physiology. To test this, we correlated lincRNA expression with ejection fraction (EF, an important indicator of heart function) and found that among the 11 modules, M4 was the only one that was positively correlated with ejection fraction (Figure 3E). We further determined the 5 most influential lincRNAs for ejection fraction using stepwise multiple linear regression (R^2^= 0.90, *P* value = 1.6e-05, Figure 3F and Table 1). Similarly, M4 was also observed to significantly correlate with left ventricular end-diastolic diameter (LVEDD, R^2^= 0.97, *P* value = 5.7e-05, Figure 3G) with 9 most influential lincRNAs identified (Table 1). These results demonstrated that M4 was highly heart-specific and could reflect particular physiological trait, providing specific biomarkers of heart failure.

**Table 1:** Stepwise multiple linear regression models with EF or LVEDD as the dependent variable

Dependent variable	Adjusted R^2^	F	Sig.	SMLR models
EF	0.90	27.19	1.6e-05	EF=45.0093-22.4776*ENSG00000247516 +25.9323*ENSG00000249816- 22.1918*ENSG00000252690+ 2.5866*ENSG00000253369+11.7599*XL OC_010594
LVEDD	0.97	50.57	5.7e-05	LVEDD=10.5798-2.4716*ENSG00000236901+ 1.8703*ENSG00000247516-2.4166*ENSG000 00252690+1.7466*ENSG00000253369-0.9979 *ENSG00000261434+1.8875 *XLOC_000027+1.4477*XLOC_038061- 7.8281*XLOC_048198+1.6696*XL OC_052037

EF: ejection fraction; LVEDD: left ventricular end-diastolic diameter; Sig.: significance; SMLR: stepwise multiple linear regression

### LincRNAs function as enhancers to regulate cardiac genes

**Figure 4:**
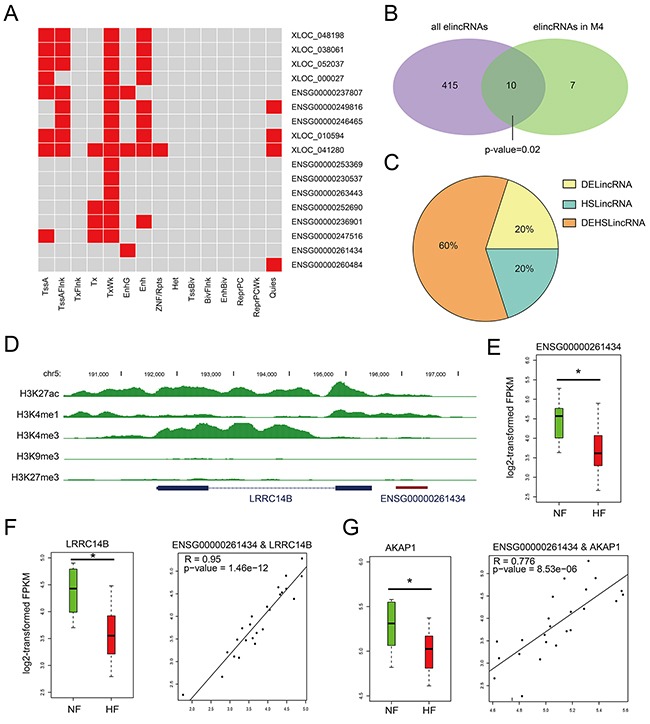
Chromatin states of lincRNAs in M4 **A.** Heatmaps showing hierarchical clustering of modules based on a certain chromatin state in lincRNAs. **B.** Venn diagram representing the significant overlap between elincRNAs and lincRNAs in M4. **C.** Pie chart representing the percentage of DElincRNAs, heart-specific lincRNAs in elincRNAs of M4. **D.** UCSC genome browser views of histone marks H3K4me1, H3K27ac, H3K4me3, H3K9me3 and H3K27me3 around ENSG00000261434. **E.** The expression levels of lincRNA ENSG00000261434 in NF controls and HF cases. **F.** The expression levels of *LRRC14B* and the expression correlation between ENSG00000261434 and *LRRC14B*. **G.** The expression levels of *AKAP1*and the expression correlation between ENSG00000261434 and *AKAP1*.

Previous studies have reported that lncRNAs can act as enhancers to regulate transcription, playing important roles in development and diseases [6, 23]. We hypothesized that a fraction of lincRNAs in M4 might be enhancer-associated lincRNAs (elincRNAs) which participated in pathogenesis of heart failure. To detect this, we first downloaded 15 chromatin state information about the left ventricle of the heart from NIH Roadmap Epigenomics Program. Consistently, as shown in Figure 4A, many lincRNAs in M4 harbored enhancer chromatin state (EnhG or Enh). We further combined H3K27ac, a characteristic enhancer activity-associated chromatin mark, to identify active elincRNAs (see Methods). We found significant enrichment of elincRNAs in M4 (10 elincRNAs, *P* value = 0.02, hypergeometric test, Figure 4B). Moreover, among these 10 elincRNAs, 8 (80%) were heart-specific and 8 (80%) were differentially expressed with an overlap of 6 (60%) elincRNAs that were both heart-specific and differentially expressed (Figure 4C).

Given that enhancer-associated lncRNAs have been characterized for their function as cis- or trans-regulatory elements [24, 25], we detected and observed that elincRNAs in M4 also possessed this feature. For example, elincRNA *ENSG00000261434* harbored peaks of histone mark H3K4me1 and H3K27ac around the promoter region (Figure 4D). Although little is directly known about its neighboring gene *LRRC14B*, the LRRC superfamily contains members which consist of one modulatory auxiliary subunit of BK channels, whose dysfunction can lead to disease in humans including high blood pressure and cardiac hypertrophy [26]. *ENSG00000261434* and *LRRC14B* showed high expression correlation (R = 0.95, Pearson's correlation) and were both expressed at significantly lower levels in HFs than NFs (*P* value < 0.05, Figures 4E and 4F), suggesting a cis-regulation of elincRNA ENSG00000261434. Moreover, we also observed its positive correlation with non-neighboring genes such as *AKAP1* (Figure 3B), both of which showed significant down-regulation in HFs (Figure 4G), implying that elincRNA *ENSG00000261434* was also likely to function in trans. Previous studies have reported that knockdown of *AKAP1* in rat cardiomyocytes could result in cellular hypertrophy [27], indicating potential regulatory role of *ENSG00000261434* in cardiomyocyte hypertrophy.

Taken together, these results suggested that lincRNAs in M4 could function as enhancers to regulate gene expression both in cis and in trans. Their dysregulation may contribute to the genesis and progress of heart failure.

### Cardiac muscle contraction is influenced by lincRNA-associated ceRNA regulation

**Figure 5:**
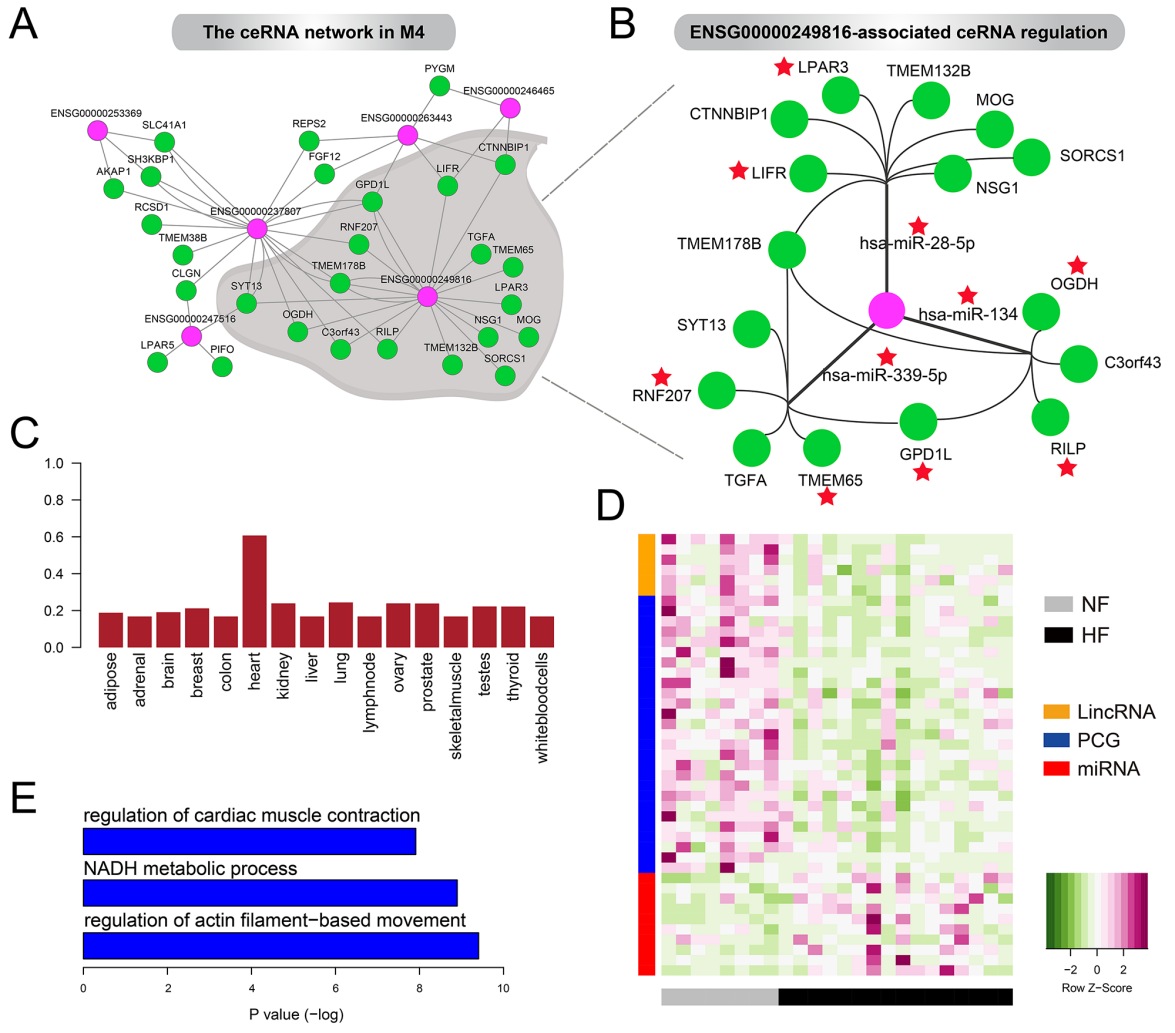
The ceRNA network in M4 **A.** The lincRNA-associated ceRNA network in M4. **B.** ENSG00000249816-associated ceRNA regulation in M4. Red stars represent the genes and miRNAs have been reported to be associated with heart development or cardiac disease including heart failure. **C.** Boxplot of the JS score of ENSG00000249816 in 16 tissues. **D.** Heatmaps showing the expression levels oflincRNAs, mRNAs and miRNAs in the ceRNA network. **E.** GO terms enriched by PCGs in the ceRNA network.

Based on the fact that lincRNAs and PCGs in M4 were co-expressed, we speculated that, apart from enhancer-like functions, lincRNAs in M4 could also control key cardiac genes by acting as competing endogenous RNAs (ceRNAs) [8, 28]. To confirm this, we identified the ceRNA crosstalk based on the predicted and experimentally validated miRNA-mRNA/lincRNA regulation and their expression levels and then constructed the ceRNA network in M4 (Figure 5A, see Methods). To ensure the reliability of the ceRNA network, we checked the expression correlations among miRNA, lincRNA and PCGs and found that the mean correlation of miRNA-lincRNA pairs, miRNA-PCG pairs and lincRNA-PCG pairs were −0.48, −0.51 and 0.62, respectively.

This ceRNA network contained 6 lincRNAs, 10 miRNAs and 27 PCGs. We found that some lincRNAs could regulate multiple genes through competing for one miRNA, such as *ENSG00000253369*, *ENSG00000246465* and *ENSG00000247516*. Also, some lincRNAs could be involved in regulating multiple ceRNAs by competing for various miRNAs. For example, *ENSG00000249816*, showing high heart specificity (Figure 5C), acted as sponges for hsa-miR-28-5p, hsa-miR-339-5p and hsa-miR-134 to modulate 16 genes such as *LIFR*, *RNF207*, *TMEM65* and *OGDH* (Figure 5B). Notably, previous studies revealed the significant loss of *LIFR* in failing heart both in humans [29] and rats [30]. Also, decreased *OGDH* activity in rat hearts after myocardial infarction has been observed [31]. Moreover, hsa-miR-339-5p has been reported to be up-regulated after human left ventricular ischemia [32] and hsa-miR-134 was an essential regulator in cardiogenesis whose increased expression was strongly associated with increased risk of mortality or heart failure [33]. Consistent with these experimental findings, we found almost all the lincRNAs (including *ENSG00000249816*) and PCGs were expressed at lower levels in HF cases compared with NF controls but miRNAs exhibited opposite patterns (Figure 5D). These expression alterations promoted us to reason that ceRNAs were closely associated with the pathogenesis of heart failure. As expected, PCGs in the ceRNA network were significantly enriched for heart systolic function, such as regulation of cardiac muscle contraction and regulation of actin filament–based movement (Figure 5E). The complex crosstalk between *ENSG00000249816* and cardiac genes, which was mediated by cardiac miRNAs, implied its crucial roles in pathology of heart failure.

Taken together, our results supported the important roles of lincRNA-mediated ceRNA regulation in M4 in the pathogenesis of heart failure, especially disturbing the left ventricular systolic function via their dysregulation.

## DISCUSSION

LincRNAs, as the dominant family of lncRNAs, play important roles in transcriptional regulation [34]. However, few studies have focused on the roles of lincRNAs in heart failure. To address this, we systematically identified and characterized the transcriptome of lincRNA, mRNA and miRNA simultaneously using RNA-seq and miRNA-seq data of 24 samples. Based on gene co-expression, 11 HF-associated lincRNA modules were identified, of which M4 showed the highest heart specificity. Moreover, we found that lincRNAs in M4 could not only function as enhancers to regulate key cardiac genes in cis or in trans but also act as ceRNAs to interfere with cardiac miRNAs, both contributing to the pathogenesis of heart failure. This work presents a comprehensive dissection of lincRNA-mediated regulation and further identifies a module (M4) closely related with the physiology and pathology of heart, providing a deeper understanding of roles of lincRNAs in heart failure.

Failing hearts are usually characterized by a global transcriptional reprogramming, leading to activation of the so-called “fetal-like” cardiac gene expressions [35]. Consistently, up-regulated PCGs identified here were enriched for many development-associated functions, such as cardiovascular system development (Figure 1G). On the other hand, down-regulated PCGs were mainly enriched for energy and metabolic process such as ATP synthesis (Figure 1G), which partly interpreted the lack of power of the failing heart to take in and/or eject sufficient blood [36]. These results suggested that the whole transcriptome constructed here could accurately reflect the physiological and pathological characteristics of the failing heart. To explore the effect of DELincRNAs on the PCG expression profile, we investigated whether expression changes of lncRNAs could influence the PCG expression through lncRNA knockdown experiments. We analyzed the knockdown data of ENSG00000226950 [37] that was down-regulated in heart failure (Supplementary Figure S6A). GSEA analysis showed significant enrichment of differentially expressed genes induced by ENSG00000226950 knockdown in the dysfunctional genes in heart failure (FDR and P value < 0.0001, Supplementary Figure S6B), suggesting significant influence on PCG expression profile by ENSG00000226950 knockdown.

We finally identified 11 HF-associated lincRNA modules and found that most modules performed specific functions with few overlaps (Figure 2B), suggesting the complexity and precision of transcriptional regulatory network with an important component of lincRNAs. For example, most PCGs in M6 were mitochondrial genes or genes involved in oxidative phosphorylation, the citric acid (TCA) cycle and respiratory electron transport. M6 contained 3 lincRNAs (including 1 DELincRNA) and 115 PCGs, of which 61 DEPCGs were all down-regulated in HF. This interesting finding was not only accordant with the results shown in Figure1G, but also confirmed the accuracy and specificity of modules identified here, considering the reduced cardiac contractility which may result from the lack of energy. To further verify this conclusion, we performed functional enrichment analysis for genes in M6 and obtained many heart and energy-associated functions such as regulation of ventricular cardiac muscle cell action potential, regulation of heart rate by cardiac conduction and cardiac muscle cell action potential involved in contraction. Despite the strong functional specificity of these modules, we also observed shared functional gene sets which were potential targets of transcription factors such as TCF3, LEF1, JUN and MYC. Notably, TCF/LEF proteins are key components of Wnt/β-catenin signaling pathway and MYC as well as JUN are Wnt target genes. We observed significant up-regulation of many key components such as *WNT9A*, *FZD7*, *CTNNB1* (β-catenin) and *LEF1*, revealing the activation of Wnt pathway in HF, which is supported by recent studies [38, 39]. Moreover, we found different modules influenced different parts of the pathway (Figure 2D), suggesting a cooperative activation of Wnt signaling pathway. These results shed additional light on regulatory diversity of Wnt pathway with lincRNAs as new regulators in heart failure.

It is known that lincRNAs were expressed in a tissue-specific manner [40]. We found that most HF-associated lincRNA modules showed high heart specificity, particularly M4, in which 71% lincRNAs were heart-specific (Figure 3C). Stepwise linear regression analysis demonstrated that lincRNAs in M4 could reflect physiological status of heart, including ejection fraction and left ventricular end-diastolic diameter. From the perspective of diagnostic and therapeutic potential, these heart-specific lincRNAs provide valuable biomarkers to monitor the status of heart and even present ideal treatment targets. Notably, more in-depth investigation of these lincRNAs is needed in peripheral blood of a much larger cohort of heart failure patients. Specifically, their association with heart failure should be confirmed after controlling for commonly measured clinical variables and standard heart failure-risk markers such as natriuretic peptides, and incremental information should be guaranteed when adding these lincRNAs to standard risk markers [41–43].

As the proposition of ceRNA hypothesis, recent studies have put effort in describing a complex miRNA-mediated post-transcriptional regulatory network, which allows indirect crosstalk between non-coding and protein-coding RNAs by competing for shared miRNAs and is essential for many important biological processes [44].We also found that lincRNAs like *ENSG00000249816* and *ENSG00000237807* in M4 could regulate heart development- or disease-associated genes through competing for various miRNAs, some of which were also reported to participate in physiology and pathology of myocardium, such as hsa-miR-339-5p, hsa-miR-494 and hsa-miR-134. Furthermore, we also observed that some genes were regulated by multiple lincRNAs via various miRNA-mediated ceRNA regulation. *GPD1L* was simultaneously modulated by *ENSG00000263443*, *ENSG00000249816* and *ENSG00000237807*, which was mediated by 4 miRNAs including hsa-miR-339-5p, hsa-miR-134, hsa-miR-508-3p and hsa-miR-216b. Notably, most previous studies revealed *GPD1L* was associated with increased risk of sudden cardiac death (SAD) in patients with coronary artery disease (CAD) [45, 46]. Our findings provided an additional layer of lincRNA-associated ceRNA regulation for *GPD1L* to influence metabolic state and electrophysiological activity of cardiomyocytes, which may lead to cardiac ischemia and heart failure. Considering that the relative abundance of ceRNAs and miRNAs is critical for ceRNA crosstalk [47], the down-regulation of lincRNAs and PCGs and up-regulation of miRNAs may represent a transformation of ceRNA crosstalk and affect regulation of ceRNA network, potentially contributing to pathology of heart. Most interestingly, the down-regulated PCGs were enriched for functions such as cardiac systolic function and energy metabolism, consistent with the reduced contractility and heart rate of failing heart, which probably demonstrated a new mechanism of heart failure. Consequently, our results not only revealed the importance of lincRNA-associated ceRNA network whose dysregulation may contribute to heart failure, but also provided new insights into the regulatory mechanism of known cardiac miRNAs and PCGs, that is, the lincRNA-mediated ceRNA regulation.

To identify important HF-associated lincRNAs in other modules, we also identified elincRNAs (Supplementary Table S2) and ceRNA-related lincRNAs (Supplementary Figure S7 and Supplementary Table S3) in the remaining modules. We obtained different numbers of elincRNAs in 12 modules except for M11 and ceRNA-related lincRNAs in 11 modules except for M8 and M10. For example, through competing for a HF-associated miRNA hsa-miR-320a, [48], lincRNA ENSG00000251628 in M6 could regulate mitochondria- and energy-associated genes such as *LRPPRC*, *FASTKD2*, *ACADSB* and *ATP11A*. These results provide valuable sources for further exploring the roles of lincRNAs in heart failure, which needs experimental validation in animals and even clinical validation in the future.

This study performed a comprehensive analysis of lincRNAs and expanded our understanding of their roles in the complex transcription regulatory network in the context of failing heart. Studies in the future should pay more attention to lincRNAs as they are potentially valuable biomarkers and treatment targets, which can also help to further uncover and complement the etiology and mechanism of heart failure.

## MATERIALS AND METHODS

### RNA-seqand miRNA-seqdata

RNA-seq and miRNA-seq data (GSE46224) [49] of 16 patients with heart failure (HFs) and 8 non-failing individuals (NFs) were downloaded from the GEO database (http://www.ncbi.nlm.nih.gov/geo/).

For RNA-seq data, the initial paired-end reads were mapped against the human genome (hg19) using Tophat (version 2.0.13) [50]. Cufflinks (version 2.2.1) [51] was used to assemble the uniquely mapped reads into transcripts for each sample and then the assemblies were merged together with Cuffmerge. Only the previously unannotated multi-exon transcripts in intergenic regions with length >= 200bp and coding probability (CP) < 0.364 were defined as novel lincRNAs, in which CP was calculated by CPAT (version 1.2.2) [52]. Read counts of known lincRNAs (obtained from GENCODE v19), novel lincRNAs and PCGs were computed using BEDTools [53]. Lowly-expressed genes (read count< 5 in more than 50% samples) were filtered out. The expression levels for each lincRNA and PCG were calculated as fragments per kilobase per million mapped reads (FPKM).

For miRNA-seq data, the miRanalyzer [54] was used to map sequence reads to miRBase v19. Read counts of each miRNA were calculated and then normalized to the total counts of sequence reads as RPMs (reads per million mapped reads). Only the miRNAs with mapped reads in more than 50% samples were retained for further analysis.

### Differential expression analysis

Differentially expressed genes were identified using read counts as input to three widely used tools, DESeq2 [55], edgeR [56] and voom-limma [57] between disease and normal samples. Only the genes with adjusted *P* values < 0.05 in at least two tools were considered as differentially expressed.

### Functional enrichment analysis

The enriched GO (Gene Ontology) terms for differentially expressed genes were identified using GOstats package [58] with FDR < 0.05.

### Identification of HF-associated lincRNA modules

We identified modules of co-expressed genes using the Weighted Gene Co-expression Network Analysis (WGCNA) package [15, 16] in R. The FPKM values of lincRNAs and PCGs in 16 HFs and 8 NFs were normalized by log2 transformation (i.e. log2(FPKM+1)) and then used as input for module detection. We used the function blockwise Module with the following parameters: power=7, minModuleSize=30, mergeCutHeight=0.1, networkType=unsigned, corType=“pearson”, minCore KME=0.8, minKMEtoStay=0.5. This resulted in 45 modules of co-expressed genes. We then assessed the correlation between module eigengenes (equivalent to the first principal component [59]) and clinical traits such as HF, sex, age, smoking and diabetes mellitus (DM). HF-associated modules were identified as those showing significant correlation (*P* value<0.05) with HF while non-significant correlation with other clinical traits. Then those modules containing dysregulated lincRNAs were defined as HF-associated lincRNA modules. Finally, we retained 11 modules containing less than 500 members.

### Tissue specificity

To evaluate the tissue specificity of genes, we downloaded RNA-seq data of 16 tissues from Human Body Map 2.0 and calculated Jensen-Shannon tissue specificity score (JS score) [60] for each gene in each tissue based on its expression level. A gene was defined as heart-specific if the JS score in heart tissue was the highest among 16 tissues and greater than 0.4.

### Stepwise multiple linear regression

We used the stepwise multiple linear regression to determine which lincRNAs in M4 mainly contributed to physiological traits of heart, such as ejection fraction and left ventricular end-diastolic diameter, based on the lincRNA expression profiles. The stepping criteria for entry and removal are based on the F test. At each step, stepwise regression reexamines each variable that previously entered in the model since it may become non-significant with other variables added to the model. The variable with the smallest non-significant F-value is removed, and the model is refitted with the other variables. This process was repeated until no more variables can be added or removed.

### Identification of enhancer-associated lincRNAs

ChIP-seq data and chromatin state (15 states) information about the left ventricle of the heart were downloaded from NIH Roadmap Epigenomics Program [61]. The 15 chromatin states were identified using ChromHMM v.1.10 [62] as previously described [63], based on the ChIP-seq data of five chromatin marks including H3K4me3, H3K4me1, H3K36me3, H3K27me3 and H3K9me3. First, the whole genome was divided into non-overlapping bins with the size of 200bp and read counts were calculated in each bin for each data set (the reads were shifted by 100bp). Through comparing ChIP-seq read counts with corresponding control read counts, each bin was then discretized into two levels (1 represents enrichment and 0 represents no enrichment) using the default discretization threshold of 1×10^−4^ in ChromHMM. A 15-state model was then trained and determined with default parameters. Next, the posterior probability of each chromatin state in each bin was calculated using the trained model. Finally, each bin was marked by the chromatin state with the maximum posterior probability.

The 15 chromatin states include TssA, TssAFlnk, TxFlnk, Tx, TxWk, EnhG, Enh, ZNF/Rpts, Het, TssBiv, BivFlnk, EnhBiv, ReprPC, ReprPCWk and Quies. (1) TssA denotes the active transcription start site (TSS), which is enriched in TSS of actively transcribed genes; (2) TssAFlnk denotes the flanking active TSS, which is enriched in immediate neighborhood of TSS of actively transcribed genes; (3) TxFlnk denotes the transcribed state at gene 5′ and 3′ ends, which is enriched at 5′ and 3′ ends of actively transcribed genes; (4) Tx denotes strong transcription, which is enriched in gene bodies of transcribed genes; (5) TxWk denotes weak transcription, which is enriched in gene bodies of transcribed genes; (6) EnhG denotes genic enhancers, which is enriched in gene bodies of transcribed genes; (7) Enh denotes enhancers, which is strongly enriched for ChIP-seq binding sites of activating enhancer TFs; (8) ZNF/Rpts denotes ZNF genes & repeats, which is enriched for ZNF genes and satellite repeats; (9) Het denotes heterochromatin, which is enriched at heterochromatin regions, centromeric and telomeric repeats; (10) TssBiv denotes the bivalent/poised TSS, which is enriched in TSS of repressed genes; (11) BivFlnk denotes the flanking bivalent TSS/Enh, which is enriched around TSS of repressed genes; (12) EnhBiv denotes the bivalent enhancer, which is enriched for ChIP-seq binding sites of activating enhancer TFs and Polycomb factors e.g. Suz12 and Ezh2; (13) ReprPC denotes repressed polycomb, which is enriched at gene bodies of repressed genes; (14) ReprPCWk denotes weak repressed polycomb, which is enriched at gene bodies of repressed genes; (15) Quies denotes quiescent regions which is enriched for no marks.

A lincRNA is defined as an enhancer-associated lincRNA if (i) the lincRNA overlaps with enhancer chromatin states (EnhG or Enh), (ii) there is at least one H3K27ac peak calculated by MACS [64] locating within +/− 5kb from the transcription start site of the lincRNA.

### Construction of the lincRNA-associated ceRNAnetwork

First, lincRNA-miRNA interaction pairs were downloaded from miRCode database [65] and miRNA-mRNA interaction pairs were downloaded from TargetScan [66] and StarBase database [67]. Since miRCode database contains lincRNAs in GENCODE v11, for newly enlisted lincRNAs in GENCODE v19, modified Smith-Waterman alignment in the miRanda algorithm was used to find seed matched miRNA target sites on them. Moreover, hypergeometric test was employed to assess the likelihood of a ceRNA pair [68].

Second, we used expressions of lincRNAs, mRNAs and miRNAs to further identify reliable ceRNA pairs to construct the ceRNA network. An edge was drawn between a lincRNA and an mRNA where (i) the lincRNA and miRNA expressions were negatively correlated with *P* value < 0.05, (ii) the mRNA and miRNA expressions were negatively correlated with *P* value < 0.05, (iii) the lincRNA and mRNA were in the same HF-associated lincRNA module, here referring to M4.

## SUPPLEMENTARY FIGURES AND TABLES








