# Supplementary material for: Dysregulated long intergenic non-coding RNA modules contribute to heart failure
Source: Oncotarget. 2016 Jul 25;7(37):59676–90. doi: 10.18632/oncotarget.10834 (PMC5312340; doi:10.18632/oncotarget.10834)
Supplement: Supplementary file 1 [file oncotarget-07-59676-s001.pdf]

Dysregulated long intergenic non-coding RNA modules contribute to heart failure

SUPPLEMENTARY FIGURES AND TABLES

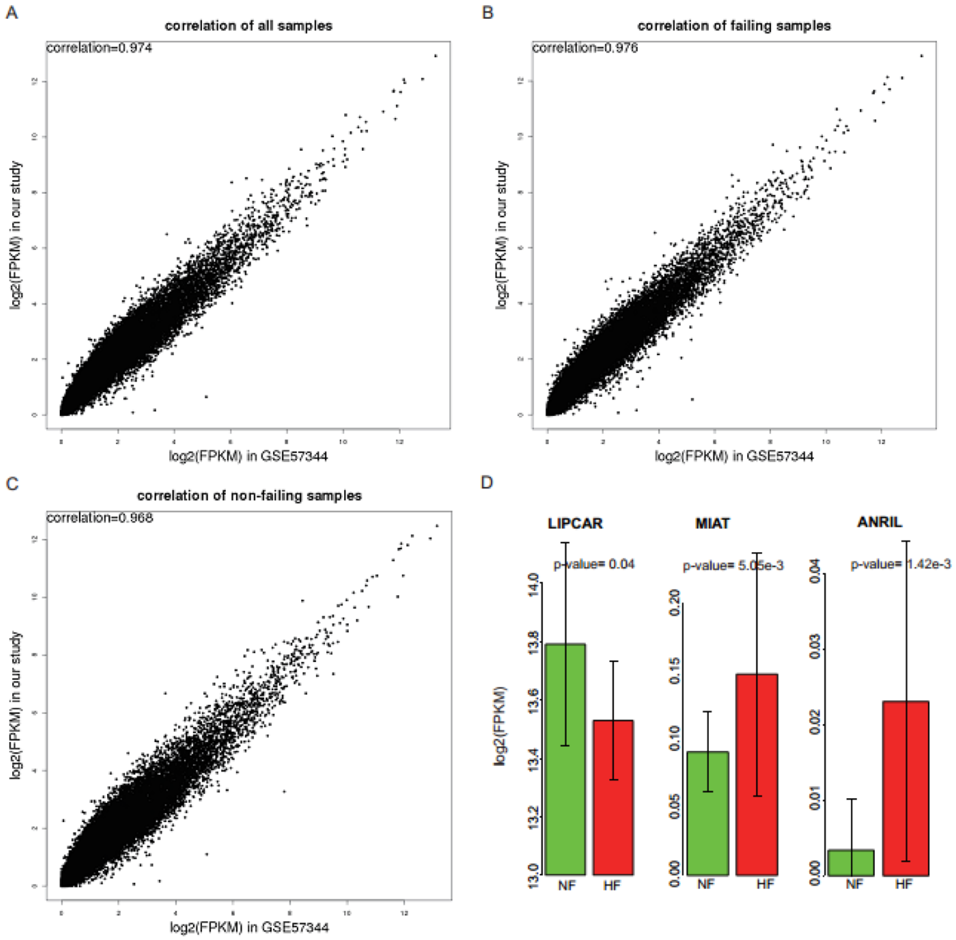

Supplementary Figure S1: Validation on the data used in our study. A-C. Comparison of mean expression levels of all genes across all samples (A) or across heart failure samples (B) or across non-failing samples (C) used in our study (Y axis) and in GSE57344 (X axis). **D.** Expression levels of three coronary artery disease-related lncRNAs including LIPCAR, MIAT and ANRIL, in heart failure samples (red bars) and in non-failing samples (green bars).

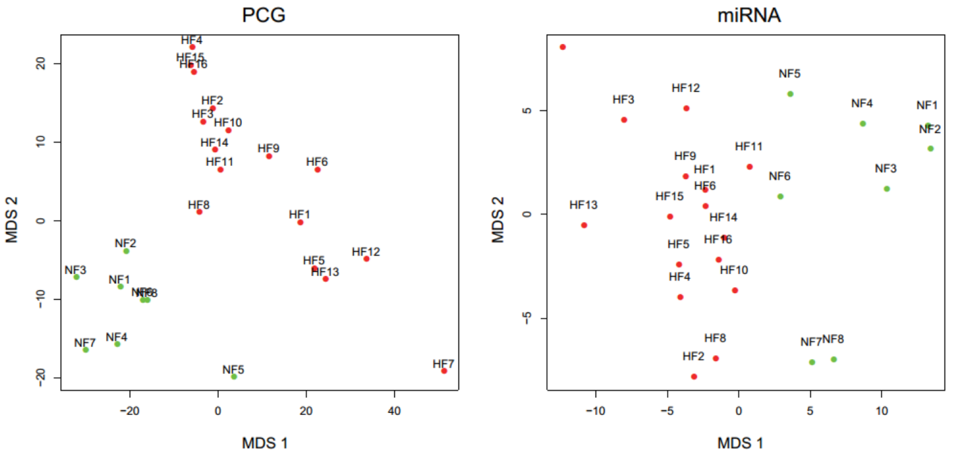

Supplementary Figure S2: MDS plot using the PCGs and miRNAs expression levels, respectively.

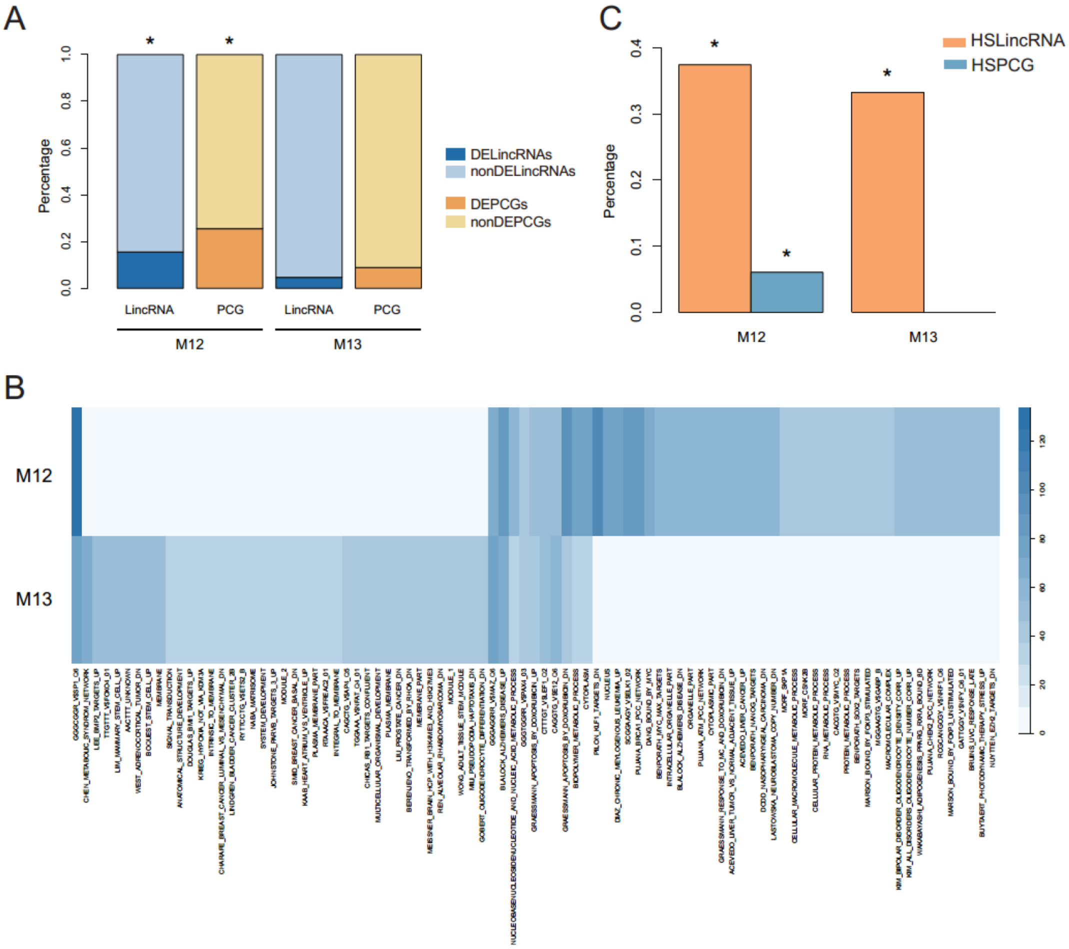

Supplementary Figure S3: A. Barplot showing the percentages of DELincRNAs and DEPCGs in M12 and M13. **B.** The functional gene sets enriched for M12 and M13. **C.** The ratios of heart-specific lincRNAs and PCGs in M12 and M13. Asterisk (*) represents significant enrichment by hypergeometric test for P < 0.05.

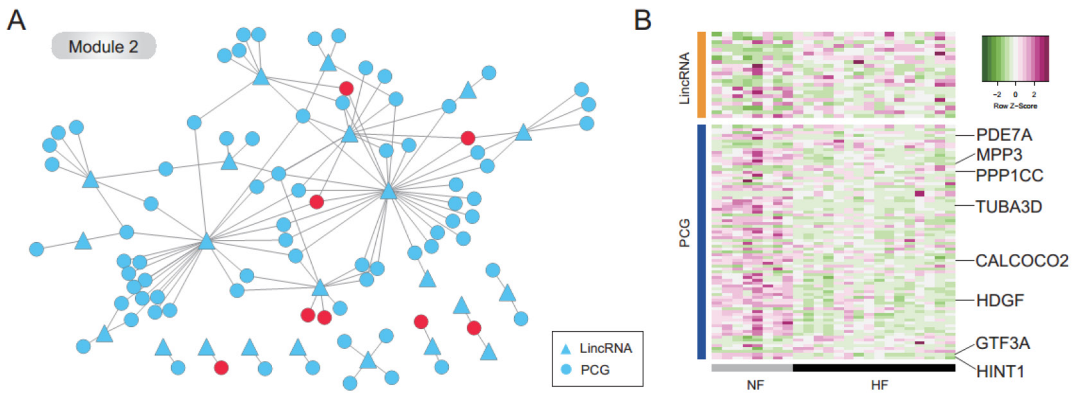

Supplementary Figure S4: A. Co-expressed network in M2 and **B.** Heatmap showing the expression levels of lincRNAs and PCGs in M2.

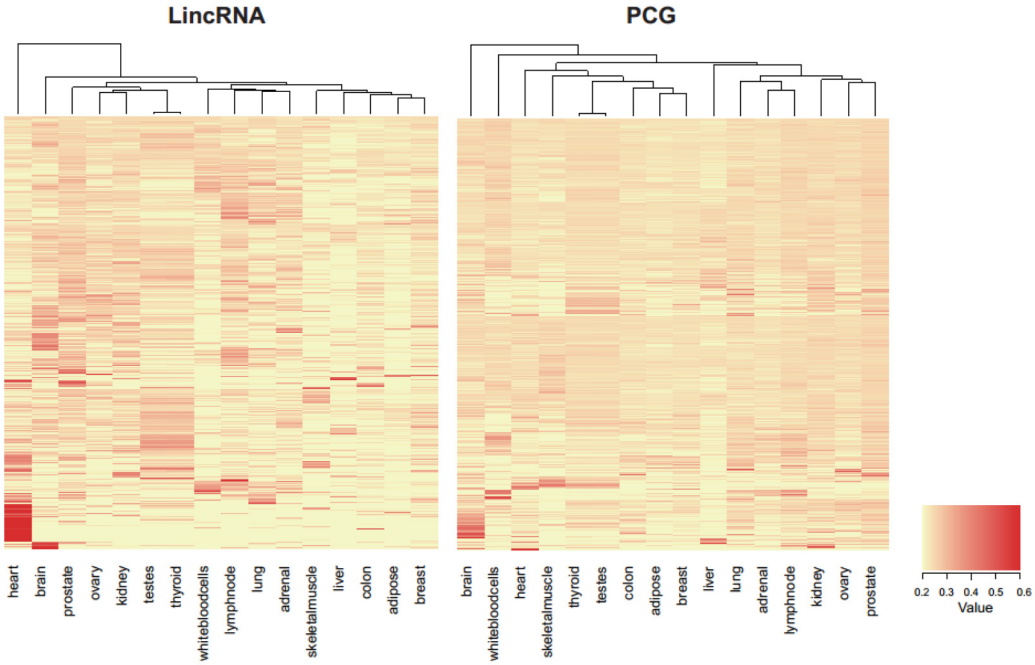

Supplementary Figure S5: Heatmaps showing the JS scores of all expressed lincRNAs and PCGs, suggesting the higher heart specificity.

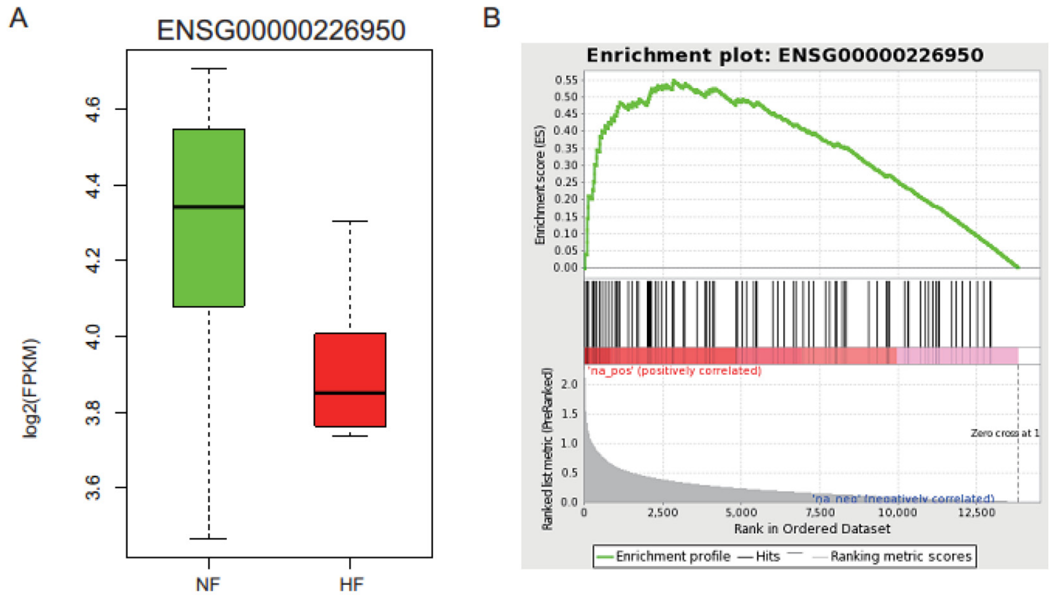

Supplementary Figure S6: A. The differential expression levels of ENSG00000226950 between non-failing samples and heart failure. **B.** The GSEA image showing the significant enrichment of differentially expressed genes induced by ENSG00000226950 knockdown in the dysfunctional genes in heart failure.

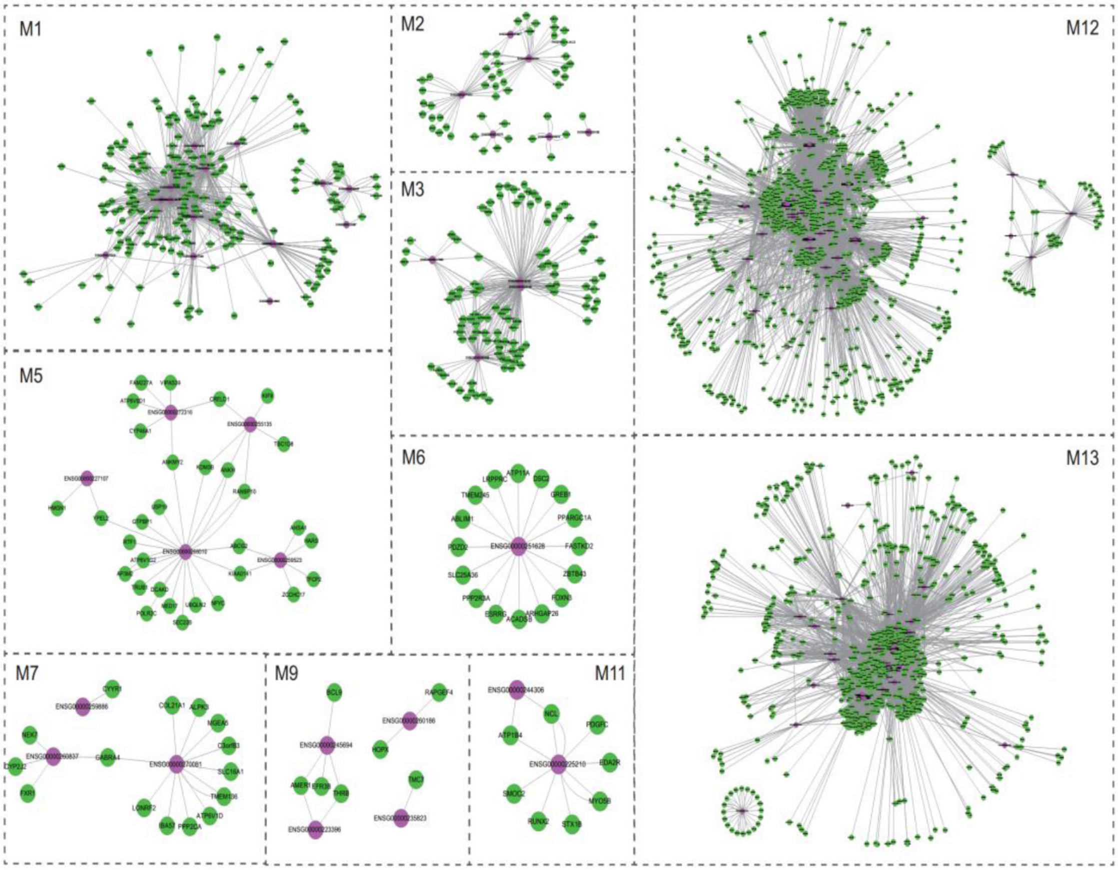

Supplementary Figure S7: The ceRNA networks identified in HF-associated modules. No ceRNAs were identified in M8 and M10.

Supplementary Table S1: Summary of demographics for samples used in this study

See Supplementary File 1

Supplementary Table S2: The elincRNAs in the HF-associated modules

See Supplementary File 2

Supplementary Table S2: The ceRNA-related lincRNAs in the HF-associated modules

See Supplementary File 3
